# Identification of a siderophore utilization locus in nontypeable *Haemophilus influenzae*

**DOI:** 10.1186/1471-2180-10-113

**Published:** 2010-04-15

**Authors:** Daniel J Morton, Elizabeth J Turman, Patrick D Hensley, Timothy M VanWagoner, Thomas W Seale, Paul W Whitby, Terrence L Stull

**Affiliations:** 1 Department of Pediatrics, University of Oklahoma Health Sciences Center, Oklahoma City, OK, 73104, USA; 2 Department of Microbiology and Immunology, University of Oklahoma Health Sciences Center, Oklahoma City, OK, 73104, USA; 3 Department of Biology, Oklahoma Christian University, Oklahoma City, OK 73136, USA

## Abstract

**Background:**

*Haemophilus influenzae *has an absolute aerobic growth requirement for either heme, or iron in the presence of protoporphyrin IX. Both iron and heme in the mammalian host are strictly limited in their availability to invading microorganisms. Many bacterial species overcome iron limitation in their environment by the synthesis and secretion of small iron binding molecules termed siderophores, which bind iron and deliver it into the bacterial cell via specific siderophore receptor proteins on the bacterial cell surface. There are currently no reports of siderophore production or utilization by *H. influenzae*.

**Results:**

Comparative genomics revealed a putative four gene operon in the recently sequenced nontypeable *H. influenzae *strain R2846 that encodes predicted proteins exhibiting significant identity at the amino acid level to proteins involved in the utilization of the siderophore ferrichrome in other bacterial species. No siderophore biosynthesis genes were identified in the R2846 genome. Both comparative genomics and a PCR based analysis identified several additional *H. influenzae *strains possessing this operon. In growth curve assays strains containing the genes were able to utilize ferrichrome as an iron source. *H. influenzae *strains lacking the operon were unable to obtain iron from ferrichrome. An insertional mutation in one gene of the operon abrogated the ability of strains to utilize ferrichrome. In addition transcription of genes in the identified operon were repressible by high iron/heme levels in the growth media.

**Conclusions:**

We have identified an iron/heme-repressible siderophore utilization locus present in several nontypeable *H. influenzae *strains. The same strains do not possess genes encoding proteins associated with siderophore synthesis. The siderophore utilization locus may enable the utilization of siderophores produced by other microorganisms in the polymicrobial environmental niche of the human nasopharynx colonized by *H. influenzae*. This is the first report of siderophore utilization by *H. influenzae*.

## Background

*Haemophilus influenzae *is a fastidious Gram-negative bacterium that is an important cause of human infections including otitis media, meningitis, and pneumonia [1]. *H*. *influenzae *is unable to synthesise protoporphyrin IX (PPIX), the immediate precursor of heme, since it lacks all enzymes in the biosynthetic pathway for the porphyrin ring [2,3]. However, most *H. influenzae *strains express a ferrochelatase which mediates insertion of iron into PPIX to form heme [2,4,5]. Thus, *H*. *influenzae *has an absolute aerobic growth requirement for an exogenous heme source or PPIX in the presence of an iron source. Since the only known niche for *H. influenzae *is humans, the organism must adapt its mechanisms of porphyrin and iron acquisition accordingly [6].

Heme is generally intracellular, in the form of hemoglobin or heme containing enzymes, and unavailable to invading microorganisms [7,8]. Extracellular hemoglobin, derived from lysed erythrocytes, is bound by the serum protein haptoglobin, and the hemoglobin-haptoglobin complex is rapidly cleared by the reticuloendothelial cells of the liver, bone marrow or spleen [9,10]. Free heme, principally derived from the degradation of methemoglobin, is bound by the serum proteins hemopexin and albumin and cleared from the circulation [7,11].

Hemoglobin and the hemoglobin-haptoglobin, heme-hemopexin, and heme-albumin complexes as well as catalase and myoglobin-haptoglobin can all be utilized by *H. influenzae *as heme sources *in vitro *[12-14]. The mechanisms underlying the utilization of these protein heme sources have been extensively studied [6,15-19].

In addition to its ability to utilize these multiple heme sources, *H. influenzae *can also grow when supplied with PPIX in the presence of an iron source in vitro. Iron sources that can be utilized under such circumstances include various iron salts as well as iron bound to the human iron-binding protein transferrin [20-24]. Utilization of iron bound to transferrin by *H. influenzae *is mediated by specific outer membrane binding proteins [25,26].

In many microbial species utilization of iron is mediated by small secreted iron binding molecules termed siderophores (generally < 1 kDa) [27,28]. Siderophores have high affinity and specificity for ferric iron, which they bind in the extracellular milieu. The siderophore-iron complex then binds to the corresponding membrane protein receptor on the cell surface as the first step in the utilization of the bound iron [27,28].

It generally has been assumed that *H. influenzae *neither produces nor utilizes siderophores as a means of iron acquisition. Evidence to support this conclusion includes the following: 1) using the universal siderophore assay of Schwyn and Neilands [29], modified to permit growth of *Haemophilus *species [30], the *H. influenzae *type b strain Eagan did not produce detectable siderophore(s) [21,30]; 2) strain Eagan was unable to utilize the exogenously supplied siderophores enterobactin, aerobactin or deferroxamine as an iron source [24]; 3) utilization of iron bound transferrin by *H. influenzae *is dependent on direct contact between the bacterial cell and transferrin, indicating that there is no release of a small iron binding molecule(s) by the bacteria [25]; 4) outer membrane proteins from iron-restricted *H. influenzae *did not react to polyclonal antisera raised against various siderophore receptor proteins from *E. coli*, whereas similar outer membrane preparations from the closely related *H. parainfluenzae *did react [24]; 5) DNA probes based on the sequence of genes encoding *E. coli *siderophore receptor proteins did not hybridize to *H. influenzae *chromosomal DNA [24]. Although these data are essentially limited to examination of type b strains they have been generally interpreted to indicate that the species *H. influenzae *in general neither produces nor utilizes siderophores.

Recently multiple genomic sequences from strains of *H. influenzae *have become available. One of these genomic sequences contains a gene cluster with significant homology to components of ferric hydroxamate uptake systems present in other bacteria. The objective of this study was to characterize these siderophore uptake gene homologs of *H. influenzae *with respect to their distribution across the species, their potential role in siderophore utilization and their regulation in response to iron and heme levels.

## Results and Discussion

### Identification of a putative siderophore utilization gene cluster in *H. influenzae*

**Figure 1:**
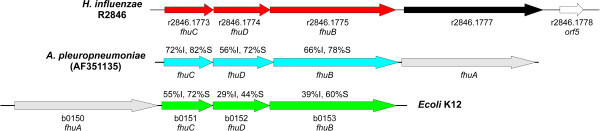
**Organization of the *H. influenzae fhu *locus and comparison of the *fhu *loci in *H. influenzae*, *A. pleuropneumoniae *and *E. coli***. The nontypeable *H. influenzae *strain R2846 *fhu *locus consists of 4 genes: 1) r2846.1777 encodes a protein with significant homology to TonB-dependent outer membrane proteins; 2) *fhuB *(r2846.1775) encodes a putative periplasmic substrate binding protein; 3) *fhuC *and *fhuD *(r2846.1773 and r2846.1774) encode putative cytoplasmic membrane permeases. Percentage identities (I) and similarities (S) are shown for pairwise comparisons of the FhuB, FhuC and FhuD proteins of nontypeable *H. influenzae *strain R2846 with the homologous proteins of *Actinobacillus pleuropneumoniae *strain 4074 (GenBack Accession No. AF351135) and *Escherichia coli *K12 substrain MG1655 (GenBack Accession No. U00096). There was no significant homology between the FhuA protein of NTHi strain R2846 and those of either *A. pleuropneumoniae *or *E. coli*. The product of *orf5 *(r2846.1778) has homology to a transposon integrase, and the gene appears not to be transcriptionally linked to the *fhu *gene cluster.

The genome sequence of the nontypeable *H. influenzae *(NTHi) isolate R2846 has recently become available [31] (Genbank Accession No. for the unfinished sequence AADO00000000). Examination of the available R2846 sequence revealed the presence of a putative siderophore uptake related gene cluster (Figure 1). This gene cluster consisted of five putative genes all apparently transcribed in the same direction. Three of these genes exhibited significant homology to genes encoding ferric hydroxamate uptake proteins of *Actinobacillus pleuropneumoniae *[32] and of *Escherichia coli *[33] (Figure 1). These three genes, designated *fhuCDB*, encode a probable ABC transport system, with *fhuB *encoding the periplasmic binding protein and *fhuCD *encoding the cytoplasmic membrane permease. In pairwise comparisons (performed using the AlignX tool of Vector NTI 10.3.0) the products encoded by *fhuC*, *fhuD *and *fhuB *of strain R2846 exhibited respectively 72%, 56% and 66% identity with the corresponding gene products from *A. pleuropneumoniae *strain 4074 (Figure 1). Corresponding figures for comparisons of the strain R2846 *fhuCDB *gene products with those of *E. coli *K12 substrain MG1655 were 55%, 29% and 39% identity respectively. These data indicate that the *fhuCBD *genes of NTHi strain R2846 constitute the ABC-transport components of a siderophore transport system.

The protein encoded by the fourth gene (locus r2846.1777) of the R2846 gene cluster did not exhibit significant homology to the FhuA protein of either *E. coli *or *A. pleuropneumoniae *(22.9% identity between FhuA of *E. coli *K12 MG1655 and R2846; 21% identity between FhuA of *A. pleuropneumoniae *strain 4074 and R2846). However, Blast searches show that the encoded protein has significant homology to TonB-dependent outer membrane proteins of other bacterial species. TonB-dependent proteins are generally associated with the uptake of iron, heme and other small molecules [34].

*Neisseria sicca*, a common nasopharyngeal commensal which rarely causes infectious disease [35], encodes a TonB-dependent receptor family protein that has the highest sequence homology to the protein encoded by r2846.1777 from *H. influenzae *(60% identity, 74% similarity). The next highest homology to r2846.1777 of R2846 (55% identity, 72% similarity) was associated with a ferric siderophore receptor produced by *Bordetella pertussis*, also a frequent colonizer of the human nasopharynx and a commonly occurring pathogen. r2846.1777 also exhibits significant amino acid identity to other uncharacterized putative TonB-dependent outer membrane proteins from a number of additional Bordetella species (*B. bronchiseptica*, *B. avium*, *B. parapertussis *and *B. petrii*), as well as *Pseudomonas*, *Burkholderia *and *Nitrosomonas *and *Acidovorax *species. These homology studies suggest that the proteins comprising the hydroxamate siderophore ABC transport system (encoded by the *fhuCDB *genes of strain R2846) may be of different origin than the putative siderophore-binding protein gene encoded by r2846.1777. The *H. influenzae *locus r2846.1777 may have originated from bacterial species known to colonize the human nasopharynx.

Thus, r2846.1777 of NTHi strain R2846 encodes a Ton-B dependent outer membrane protein of unknown function. However, it is likely, based on its proximity to genes encoding proteins showing significant identity at the amino acid level to known siderophore associated periplasmic transport systems, that r2846.1777 encodes a siderophore-binding outer membrane binding protein. However, since the product of r2846.1777 exhibits low homology with characterized FhuA proteins and since, to date, we have been unable to construct a mutant in r2846.1777 for phenotypic analyses we will use the designation r2846.1777 in the following discussions of this putative gene and its encoded protein.

The *fhu *gene cluster of NTHi strain R2846 is similarly arranged to those of *A. pleuropneumoniae *in that the putative receptor encoding gene (r2846.1777) is located downstream of *fhuCDB*, in contrast to the gene arrangement in *E. coli *where the outer membrane protein-encoding gene (*fhuA*) is upstream of the other three genes. The gene arrangement seen in both NTHi strain R2846 and *A. pleuropneumoniae*, has also been reported for a third representative of the family *Pasteurellaceae*, namely *H. parasuis *[36].

Blast searches demonstrate that the fifth gene of the gene cluster (designated *orf5 *in Figure 1) identified in NTHi strain R2846 exhibits significant homology to an internal fragment of a transposon integrase (data not shown). Bioinformatic analyses indicate that *orf5 *is not transcriptionally linked to the *fhu *gene cluster, and thus *orf5 *is not a major focus of this study.

Although a putative siderophore transport system was identified in NTHi strain R2846, no genes with significant homology to known siderophore biosynthetic genes were detected in the R2846 genomic sequence. The expression of receptor proteins that recognize siderophores produced by other microorganisms (termed xenosiderophores) is a well established characteristic of many bacterial species. These include members of the *Pasteurellaceae*, as well as most enteric species, *Bordetella *species, Pseudomonads and the mycobacteria [24,36-41]. Possession of a system(s) allowing utilization of xenosiderophores may be of benefit to NTHi strains in the complex polymicrobial environment of the human nasopharynx that this organism colonizes.

### Species distribution of the *fhu *genes

Since an apparent siderophore uptake associated locus was detected in the genomic sequence of NTHi strain R2846 further analyses were performed to determine how widely this locus is distributed within the species.

Initially Blast searches were performed against fourteen NTHi genomic sequences (four complete, eleven in process of assembly) available at the National Center for Biotechnology Information [42], as well as three *H. influenzae *genomic sequences available at the Wellcome Trust Sanger Institute [43]. Of these seventeen total genomic sequences, five contained a locus homologous to the *fhu *locus of strain R2846 (Table 1). The five strains containing a *fhu *gene cluster were all nontypeable strains and were isolated from various niches; the six total strains identified as possessing the *fhu *locus were respectively isolated from: 1) a middle ear effusion from a child with acute otitis media (strain R2846), 2) middle ear effusions from children with chronic otitis media (both strains PittEE and PittHH), 3) the nasopharynx of healthy children (both strains 22.4-21 and R3021) and 4) an adult with chronic obstructive pulmonary disease (strain 7P49H1). The *fhu *negative strains also contained examples of strains associated with each of the above listed disease states/niches. In addition the *fhu *negative strains include a single strain isolated from the external ear canal of a child with otorrhea (PittGG), a nontypeable strain isolated from the blood of a patient with meninigitis (R2866), a tybe b strain isolated from a patient with meningitis (strain 10810) and two isolates of *H. influenzae *biogroup aegyptius associated with an invasive infection termed Brazilian purpuric fever [44]. No correlation between disease state/niche and presence of the *fhu *genes was evident.

**Table 1:** Presence of *fhu *genes in sequenced *H. influenzae *strains

Strain	**Source**^**a**^	**Type**^**b**^	**GenBank Accession No.**^**c**^	***fhu *locus**^**d**^
Rd KW20	-	nt	L42023.1	No

86-028NP	NP AOM	nt	CP000057.2	No

PittEE	MEE COM	nt	CP000671.1	Yes

PittGG	Ext. Ear Ott.	nt	CP000672.1	No

22.1-21	NP Healthy	nt	AAZD00000000	No

22.4-21	NP Healthy	nt	AAZJ00000000	Yes

3655	MEE AOM	nt	AAZF00000000	No

6P18H1	Adult COPD	nt	AAWW00000000	No

7P49H1	Adult COPD	nt	AAWV00000000	Yes

PittAA	MEE COM	nt	AAZG00000000	Yes

PittHH	MEE COM	nt	AAZH00000000	No

PittII	MEE COM	nt	AAZI00000000	No

R2866	BLD	nt	AADP00000000	No

R3021	NP Healthy	nt	AAZE00000000	Yes

10810	Meningitis	b	na	No

F3031	BPF Clone	aegyptius	na	No

F3047	BPF Clone	aegyptius	na	No

^a ^Site and/or disease state from which strain isolated; NP, nasopharynx, AOM, acute otitis media; MEE, middle ear effusion; COM, chronic otitis media; Ext. Ear Ott, Isolate from external ear in patient with ottorhea; Healthy, Healthy child; COPD, chronic obstructive pulmonary disease; BLD, blood. No source is given for Rd KW20 since this a laboratory strain that has been passaged multiple times since its original isolation [63,74,75].

^b ^nt, nontypeable strain; b, type b strain; aegyptius, *H. influenzae *biogroup aegyptius.

^c ^GenBank Accession Numbers beginning with L or C denote completed genomic sequence, those beginning with AA denote sequences in process of assembly. na, not available (no GenBank accession numbers are available, sequences are accessible at the Wellcome Trust Sanger Institute [43]).

^d ^Yes, *fhu *locus is present; No, *fhu *locus is absent.

As is the case for NTHi strain R2846, none of the *H. influenzae *genomic sequences analyzed above contained genes with homology to known siderophore biosynthetic genes.

In addition to the above in silico analyses of sequenced *H. influenzae *genomes a PCR based survey of selected strains from a laboratory collection of *H. influenzae *isolates which had been previously characterized by the electrophoretic mobility of 15 metabolic enzymes [45] was performed. Thirty-nine strains representing 39 different electrophoretic types (ETs) were used in this study; four of these strains were type b strains and 35 were serologically nontypeable. In addition to characterization by ET these strains were previously characterized by biotype, and representative strains of each of the five biotypes were analyzed (Table 2). PCR assays for the presence of each gene in the *fhu *locus in each strain were repeated at least twice. Of the four type b strains tested, none were positive for the presence of any gene in the *fhu *locus (Table 2). In considering strains by biotype, all of the tested strains of biotypes I, IV and V were negative for the presence of all genes in the *fhu *locus (Table 2). Of six strains of biotype II, one strain (HI1374) was positive for the presence of *fhuCDB *and r2846.1777 but was negative for the presence of *orf5 *(although in at least one of several separate assays the *orf5 *primers were weakly positive with strain HI1374). Of 21 strains of biotype III, six strains were consistently positive for the presence of all five genes, ten strains were positive for the presence of at least four genes, and one strain (HI1389) was consistently positive for the presence of three genes. Four of the total 21 biotype III strains tested were consistently negative for the presence of all five genes. Negative results for one of the genes of the *fhu *operon in some strains may result from minor DNA sequence variations leading to inefficient primer binding and probably do not reflect absence of the gene. For example strain HI1380 was negative by PCR for the presence of *fhuB*, but in growth curve assays was able to utilize ferrichrome as a heme source indicating that *fhuB *is likely to be present (see data in Growth studies section below). Strains that consistently gave positive results for at least three of the five genes are designated as being positive for the presence of the *fhu *gene cluster.

**Table 2:** Presence of *fhu *genes in unsequenced *H. influenzae *strains

				**Gene**^e^				
**Template** **Strain**^**a**^	**Source**^**b**^	**ET**^**c**^	**BT**^**d**^	**r2846.** **1777**	***fhuD***	***fhuB***	***fhuC***	***orf5***

HI678 (b)	INV	2	I	No	No	No	No	No

HI1408 (nt)	CSF	68	I	No	No	No	No	No

HI1409 (nt)	EAR	69	I	No	No	No	No	No

HI1416 (nt)	EAR	76	I	No	No	No	No	No

HI1424 (nt)	EAR	84	I	No	No	No	No	No

HI673 (b)	INV	47	II	No	No	No	No	No

HI679 (b)	CSF	15	II	No	No	No	No	No

HI1374 (nt)	CSF	26	II	Yes	Yes	Yes	Yes	No

HI1375 (nt)	EAR	27	II	No	No	No	No	No

HI1400 (nt)	EAR	60	II	No	No	No	No	No

HI699 (b)	INV	46	III	No	No	No	No	No

HI1372 (nt)	BLD	12	III	Yes	Yes	Yes	Yes	Yes

HI1373 (nt)	EAR	13	III	No	No	No	No	No

HI1376 (nt)	EAR	29	III	Yes	Yes	Yes	Yes	Yes

HI1377 (nt)	EAR	30	III	Yes	Yes	Yes	Yes	Yes

HI1380 (nt)	BLD	35	III	Yes	Yes	No	Yes	Yes

HI1381 (nt)	BLD	36	III	Yes	Yes	Yes	Yes	Yes

HI1382 (nt)	EAR	37	III	Yes	Yes	Yes	Yes	No

HI1383 (nt)	EAR	38	III	No	Yes	Yes	Yes	Yes

HI1384 (nt)	EAR	39	III	Yes	Yes	Yes	Yes	Yes

HI1385 (nt)	EAR	40	III	Yes	Yes	Yes	Yes	No

HI1386 (nt)	BLD	41	III	Yes	Yes	Yes	Yes	No

HI1387 (nt)	EAR	42	III	Yes	Yes	Yes	Yes	No

HI1389 (nt)	EAR	44	III	Yes	Yes	Yes	No	No

HI1390 (nt)	BLD	45	III	Yes	Yes	Yes	Yes	No

HI1397 (nt)	EAR	57	III	Yes	Yes	Yes	Yes	No

HI1399 (nt)	EAR	59	III	No	No	No	No	No

HI1420 (nt)	CSF	80	III	Yes	Yes	Yes	No	Yes

HI1422 (nt)	BLD	82	III	No	No	No	No	No

HI1423 (nt)	EAR	83	III	Yes	Yes	Yes	Yes	No

HI1425 (nt)	EAR	85	III	Yes	Yes	Yes	Yes	Yes

HI1410 (nt)	EAR	70	IV	No	No	No	No	No

HI1417 (nt)	EAR	77	IV	No	No	No	No	No

HI1428 (nt)	BLD	92	IV	No	No	No	No	No

HI1429 (nt)	BLD	93	IV	No	No	No	No	No

HI1430 (nt)	BLD	94	IV	No	No	No	No	No

HI1378 (nt)	EAR	31	V	No	No	No	No	No

HI1379 (nt)	EAR	32	V	No	No	No	No	No

HI1388 (nt)	EAR	43	V	No	No	No	No	No

^a ^nt, nontypeable strain; b, type b strain

^b ^Site from which strain derived. INV, invasive disease, but site of isolation unrecorded; CSF, cerebrospinal fluid; BLD, blood.

^c ^ET, Electrophoretic type

^d ^BT, biotype

^e ^Gene tested for in polymerase chain reaction.

Combining the in silico analysis of sequenced isolates and the PCR analysis of additional strains these data indicate that the *fhu *locus is limited in distribution to nontypeable strains of *H. influenzae*. None of the five type b strains analyzed (the sequenced isolate 10810 and four additional strains analyzed by PCR) contained the locus. Among the NTHi strains the locus appears to be predominately restricted to those of biotype III; of the 18 strains that were positive for presence of the *fhu *locus by PCR, 17 were of biotype III and one was of biotype II. In considering the sequenced isolates that contained the *fhu *genes strain R2846 is a biotype III strain and strain R3021 is a biotype II strain (no biotype has been reported for the remaining *fhu *positive sequenced strains).

In contrast to the clear association with biotype III strains presence of the *fhu *locus cannot be associated with any particular disease state/niche since strains containing the *fhu *locus have been isolated from multiple sites (Tables 1 and 2).

A potential siderophore utilization locus has been identified in NTHi that appears to be limited to strains of biotype II and biotype III, and to predominantly occur in biotype III strains.

### Growth studies

Since some *H. influenzae *strains possess an apparent siderophore utilization associated gene locus but lack the corresponding siderophore biosynthesis genes, the ability of such strains to utilize an exogenously supplied siderophore was determined. Since homologous genes in *E. coli *and *A. pleuropneumoniae *are associated with the utilization of ferrichrome [33,46], growth assays were performed with ferrichrome as the sole iron source.

**Figure 2:**
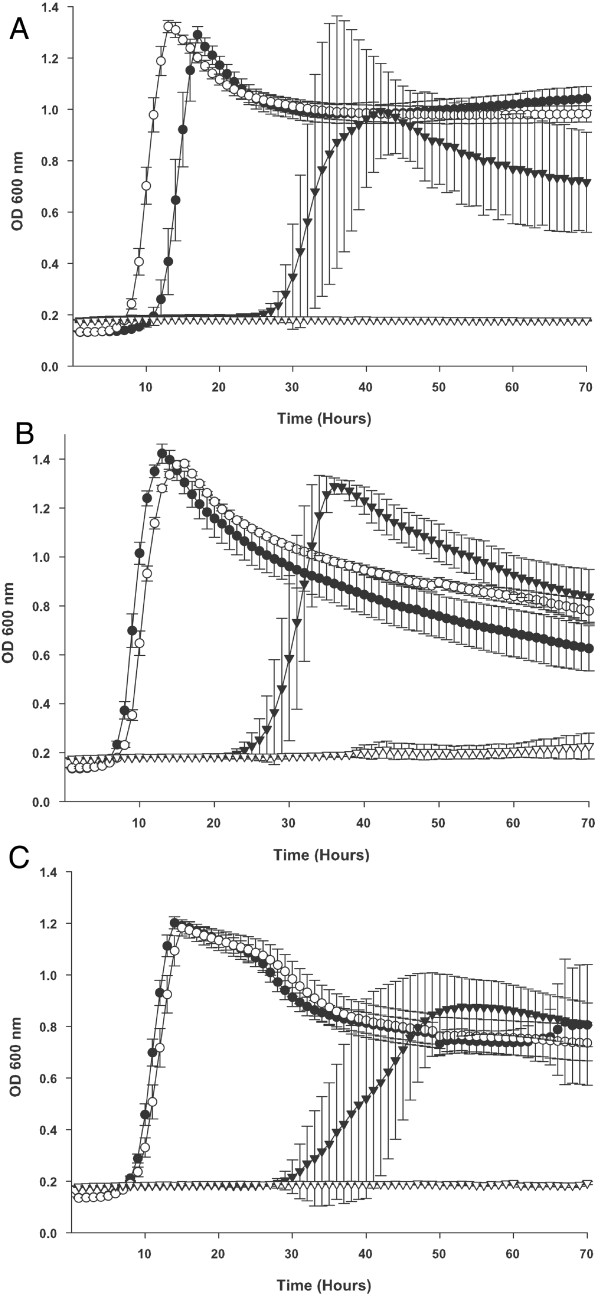
**Growth of *H. influenzae *strains R2846, HI1380 and HI1390 and their corresponding isogenic *fhuD *insertion mutant derivatives with ferric ferrichrome as the sole iron source**. Growth of all strains is in either hdBHI supplemented with heme as the sole heme and iron source or in hdBHI supplemented with protoporphyrin IX as a porphyrin source, EDDA to chelate free iron and ferric ferrichrome as the sole iron source. (A) Wildtype strain R2846 with heme at 10 μg ml^-1 ^(solid circles) and with ferric ferrichrome at 200 μM (solid triangles). The *fhuD *insertion mutant strain HI2128 with heme at 10 μg ml^-1 ^(open circles) and with ferric ferrichrome at 200 μM (open triangles). (B) Wildtype strain HI1380 with heme at 10 μg ml^-1 ^(solid circles) and with ferric ferrichrome at 200 μM (solid triangles). The *fhuD *insertion mutant strain HI2131 with heme at 10 μg ml^-1 ^(open circles) and with ferric ferrichrome at 200 μM (open triangles). (C) Wildtype strain HI1390 with heme at 10 μg ml^-1 ^(solid circles) and with ferric ferrichrome at 200 μM (solid triangles). The *fhuD *insertion mutant strain HI2132 with heme at 10 μg ml^-1 ^(open circles) and with ferric ferrichrome at 200 μM (open triangles). Results are mean ± SD for quintuplicate results from representative experiments.

Figure 2A shows that NTHi strain R2846 can readily grow when supplied with ferric ferrichrome as the sole iron source. Several additional strains whose genomes have been sequenced and which lack the *fhu *operon were also assessed for their ability to utilize ferric ferrichrome as the sole iron source; none of the following strains were able to utilize ferric ferrichrome: Rd KW20, type b strain 10810, NTHi strain 86-028NP and the NTHi strain R2866 (data not shown).

Having established that strain R2846 can utilize ferric ferrichrome as a sole iron source we set out to determine if the *fhu *gene cluster was involved in the utilization of this iron source. An insertional mutation within the coding sequence of *fhuD *was successfully constructed as described in the methods section and a mutation derivative of strain R2846 was designated HI2128. Figure 2A shows that strain HI2128 was unable to grow when supplied with ferric ferrichrome as the sole iron source. The same mutation did not significantly impair the utilization of heme alone (Figure 2A) or either ferric citrate nor ferrous ammonium sulphate in the presence of PPIX (data not shown), indicating that the defect is specific for the ferrichrome molecule rather than impacting the acquisition of the iron moiety or of PPIX.

In addition to strain R2846 the *fhuD *insertional mutation was introduced into two strains that were positive for the presence of the *fhu *gene cluster as determined by PCR analyses (Table 2); the two additional strains into which the *fhuD *mutation was introduced were HI1380 and HI1390 and correctly constructed mutants of each were identified and designated HI2131 and HI2132 respectively. Both strains HI1380 and HI1390 were able to utilize ferric ferrichrome as an iron source while neither of the corresponding *fhuD *insertion mutants, HI2131 and HI2132, were able to do so (Figures 2B and 2C). Similarly to the data reported for NTHi R2846 neither of the mutant strains were impacted in their ability to utilize other heme and iron sources (Figures 2B and 2C).

These data demonstrate that *H. influenzae *strains containing the *fhu *operon are able to utilize at least one exogenously supplied siderophore, ferrichrome, as an iron source. Ferrichrome is synthesized by members of the fungal genera *Aspergillus*, *Ustilago *and *Penicillium*, and may not represent a readily available iron source in the human nasopharynx. Thus, ferrichrome may not represent the ideal substrate for the *fhu *locus of *H. influenzae *which would be utilized relatively inefficiently and this fact may be reflected in the long lag time observed for growth in ferrichrome. However, the *fhuBCDA *system may function more efficiently to transport other xenosiderophores produced by other microorganisms and further investigations will aim to address this issue.

### Iron/heme repression of transcription of the *fhu *genes

**Figure 3:**
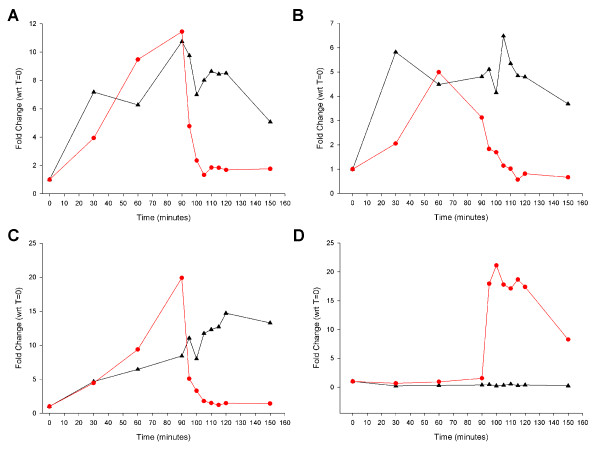
**Repression or induction of transcription of genes in response to addition of iron and heme**. Fold changes in expression of four genes in *H. influenzae *strain R2846 over the course of 150 minutes of growth under two different growth conditions. Strain R2846 was grown in either: 1) medium that was restricted for iron and heme for the duration of the experiment (black triangles) or 2) medium that was restricted for iron and heme up to 90 minutes at which point iron and heme were added to fully supplement the medium (red circles). Results are shown for r2846.1777 (A), *fhuC *(B), *hxuC *(C) and *adhC *(D).

Since the genes of the identified *fhu *gene cluster are involved in acquisition of iron the potential role of iron and heme (FeHm) in the regulation of transcription of the genes was determined; since *fhuC *and r2846.1777 are respectively the first and last genes in the putative operon transcriptional analysis within the operon was limited to these two genes. At the same time transcript levels of the two following genes were also determined: 1) *hxuC*, encoding a heme-hemopexin acquisition associated outer membrane protein [17,47] and 2) *adhC*, encoding a glutathione-dependent alcohol dehydrogenase [48]. These latter two genes were selected since they represent examples of genes the transcription of which are repressible by FeHm (*hxuC*) and inducible by FeHm (*adhC*) in multiple *H. influenzae *strains [49,50]. Two flasks containing FeHm-restricted media were inoculated with strain R2846 and incubated at 37°C with shaking. Samples (500 μl) were taken from both flasks at 30 minute intervals over the first 90 minutes of incubation for RNA isolation and quantitative-PCR (Q-PCR) analysis. After this first 90 minute interval FeHm (0.5 mM FeCl_3_, 10 μg/ml heme) was added to one of the two flasks and samples were removed at 5 minute intervals from both flasks for RNA isolation and Q-PCR. Figure 3 shows the transcript profile for all four target genes over the 150 minute total duration of the experiment. For the three genes *fhuC*, r2846.1777 and *hxuC *transcript levels in both flasks rose steadily over the first 90 minutes of the experiment. In the flask to which FeHm was added at 90 minutes transcript levels of all three genes fell substantially within 5 minutes following addition of FeHm and continued to fall thereafter, reaching a plateau at between 15 and 25 minutes following addition of FeHm (Figure 3). In contrast in the flask which remained iron restricted for the duration of the experiment transcript levels of *fhuC*, r2846.1777 and *hxuC *remained elevated through the entire 150 minute experiment. Transcript levels of *adhC *did not change in either flask during the first 90 minutes of incubation but rose rapidly following the addition of FeHm reaching a plateau within 10 minutes (Figure 3D). These data demonstrate that expression of the *fhu *operon in strain R2846 is repressible by high levels of FeHm, consistent with a role for this operon in the acquisition of siderophore bound iron. Iron and heme acquisition associated proteins of NTHi, including *hxuC*, have also been shown to be transcribed *in vivo *during clinical disease [51], indicating the importance of iron and heme acquisition in the disease process.

## Conclusions

Our data demonstrate that the *H. influenzae *strains containing the *fhu *operon are able to utilize at least one exogenously supplied siderophore, ferrichrome, as an iron source. However, these strains lack the genes encoding the biosynthesis of ferrichrome. Ferrichrome is a naturally occurring, prototypic compound of the hydroxamate class of siderophores. It remains unclear whether ferrichrome itself, or another biologically produced ferric-hydroxamate, is actually utilized in vivo by *fhu*-positive strains of *H. influenzae*.

Several additional points relevant to this newly identified siderophore utilization operon of *H. influenzae *deserve comment. 1) In addition to *H. influenzae*, other opportunistic pathogens commonly resident in the oropharynx also contain a functional hydroxamate siderophore utilization operon but do not encode genes for the production and export of hydroxamate siderophores. Examples of such microorganisms include *Staphylococcus aureus *[52], *Streptococcus pneumoniae *[53], *Neisseria meningitidis *[54] and the yeast, *Candida albicans *[55,56]. This observation suggests that the acquisition of a complete uptake system for the utilization of hydroxamate xenosiderphores is adaptive for *H. influenzae *as it appears to be for other residents of the human oropharynx.

2) The occurrence in the oropharynx of multiple species which are capable of utilizing, but not synthesizing, ferric-hydroxamates as iron sources implies that one or more microbial sources producing this siderophore class are likely to occur in this niche. This observation supports the contention that presence of the *fhu *locus is potentially advantageous to those NTHi strains that possess these genes.

3) Bacteria residing in the human oropharynx and possibly other sites, such as the lung, are the most plausible microbial source of ferrichrome-like compounds available to *H. influenzae*. Ferrichrome is known to be produced by certain filamentous fungi but these species do not occur in the human body. Approximately 700 species of bacteria exist in the oropharynx of normal adult humans and over 300 bacterial species are present in dental plaque. The opportunity for the occurrence of hydroxamate siderophores in the oropharynx appears likely in this bacteria-laden, iron-limited environment. While many of the bacterial species colonizing the oropharynx are likely to be unable to synthesize hydroxamate siderophores, multiple species are known to do so, including *Pseudomonas aeruginosa *[57], *Burkholderia cenocepacia *[58] and *B. pertussis *[59]. This observation suggests that ferric hydroxamates are likely to be available to nontypeable *H. influenzae *resident within the nasopharynx.

Lastly, nontypeable strains of *H. influenzae *are known to be frequent participants in polymicrobial lung colonization and lung infections involving *S. aureus*, *S. pneumoniae*, *P. aeruginosa *and Burkholderia species as well as other bacterial species known to produce and/or utilize hydroxamate siderophores [60,61]. Such polymicrobial infections occur in the lungs of cystic fibrosis patients, in patients with chronic obstructive pulmonary disease, as well as at sites in immunocompromized patients. As a subject for future studies, we postulate that expression of a functional *fhu *operon in nontypeable strains of *H. influenzae *is likely to afford a growth advantage by selectively increasing iron acquisition from ferric-hydroxamates produced by other bacteria in the mixed commensal environments of the healthy nasopharynx and within sites of polymicrobial infection.

## Methods

### Bacterial strains and growth conditions

NTHi strain R2846 (strain 12) is a clinical isolate from the middle ear of a child with acute otitis media [62]. Strain Rd KW20 is the originally sequenced *H. influenzae *isolate [63] and was obtained from the ATCC. NTHi strain R2866 is a clinical isolate from the blood of an immunocompetent child with clinical signs of meningitis subsequent to acute OM [64]. NTHi strain 86-028NP is a minimally passaged clinical isolate from a pediatric patient who underwent tympanostomy and tube insertion for treatment of chronic otitis media [65,66]. *H. influenzae *type b strain 10810 was isolated from an individual with meningitis and its genome has been completely sequenced [43]. Additional *H. influenzae *strains are as shown in Table 2 and correspond to strains previously characterized by electrophoretic mobility of 15 metabolic enzymes [45]. *H. influenzae *were routinely maintained on chocolate agar with bacitracin at 37°C. When necessary, *H. influenzae *were grown on brain heart infusion (BHI) agar supplemented with 10 μg ml^-1 ^heme and 10 μg ml^-1 ^β-NAD (supplemented BHI; sBHI) and the appropriate antibiotic(s). Heme-deplete growth was performed in BHI broth supplemented with 10 μg ml^-1 ^β-NAD alone (heme-deplete BHI; hdBHI). Iron restriction in growth curves was achieved by the addition of 100 μM ethylenediamine di-*o*-hydroxyphenyl acetic acid (EDDA) to media when specified. EDDA was freed from contaminating iron prior to use as described by Rogers [67]. Iron restriction for expression experiments was achieved by the addition of 150 μM deferroxamine to media when specified. Spectinomycin was used at 200 μg ml^-1 ^when required for growth of *H. influenzae*.

### Porphyrin and iron sources

Hemin and PPIX were purchased from Sigma. Stock heme solutions were prepared at 1 mg ml^-1 ^hemein 4% v/v triethanolamine as previously described [68]. Stock PPIX solutions were prepared at 1 mg ml^-1 ^in water and sterilized by autoclaving prior to use.

Ferrichrome was purchased from Sigma. Ferrichrome was saturated with ferric iron by mixing with equimolar amounts of ferric citrate and incubating a room temperature for 2 hours prior to use in growth curves.

### DNA methodology

Restriction endonucleases were obtained from New England Biolabs (Beverly, MA). Genomic DNA was isolated using the DNeasy Tissue Kit (Qiagen, Valencia, CA). Plasmid DNA was isolated using Wizard Plus Minipreps DNA purification system (Promega, Madison, WI) according to the manufacturer's directions. Sequencing of double-stranded template DNA was performed by automated sequencing at the Recombinant DNA/Protein Resource Facility, Oklahoma State University, Stillwater, OK, USA. Oligonucleotides were synthesized by Operon.

### PCR analyses of *fhu *locus distribution in *H. influenzae*

Primers were designed for use in the polymerase chain reaction (PCR), based on the available sequence of the *fhu *gene cluster in NTHi strain R2846, to survey for the presence of the five genes comprising the locus. The sequences of the primers comprising each of the five primer pairs are shown in Table 3. PCRs were performed in a 50 μl volume using 100 ng of the appropriate chromosomal DNA as template, and the reactions contained 2 mM MgCl_2_, 200 μM each deoxynucleoside triphosphate (New England Biolabs), 10 pmol of each primer and 2 U of FastStart Taq DNA Polymerase (Roche, Indianapolis, IN, USA). PCR was carried out for 30 cycles, with each cycle consisting of denaturation at 95°C for 1 min, annealing for 1 min at the appropriate temperature and primer extension at 72°C for 1 min with one final extension of 30 min. Annealing temperatures were 58°C for the primer pair directed at *fhuA *and 57°C for the other four primer pairs.

**Table 3:** Primers used in PCR survey for presence of *fhu *genes

**Primer**^**a**^	Sequence 5' to 3'
R2846.1773(fhuC)_F	GGTTCGATTTCGTTGGACG

R2846.1773(fhuC)_R	GACGATTTGCTGTGCGTC

R2846.1774(fhuD)_F	CAGTGGGCGATATGCAAAG

R2846.1774(fhuD)_R	GTTTGGCGAGTTCGGTG

R2846.1775(fhuB)_F	GCGCAAAACCATGTCGC

R2846.1775(fhuB)_R	GTCGGGAAACTGAGTTGC

R2846.1777(OMP)_F	CGTCACTTTATCCAGCATCAG

R2846.1777(OMP)_R	GATAGCGTATCGGAAGC

R2846.1778(orf5)_F	GCTTAGCACGCAGTACG

R2846.1778(orf5)_R	CTCCTCTGTGTATTAAATTCC

^a ^Primer pairs used to assay for each gene.

### Construction of *fhuD *insertion mutants

An insertion mutation of *fhuD *was constructed as follows. A pair of primers was designed for use in the PCR, based on the available NTHi strain R2846 genomic sequence, to amplify an 848-bp region internal to the *fhuD *gene. Primers were designated FhuC-dnA and FhuC-dnB and had the respective sequences 5'-**GGATCC**CACTGCTCGGAATGACC-3' and 5'-**AAGCTT**CGTGCAGTAAGCCATCG-3' (those portions of the primers shown in boldface represent restriction sites engineered into the primers for directional subcloning; the engineered restriction sites were not utilized as part of this study). The PCR was performed as described above using 100 ng of strain R2846 chromosomal DNA as template and with annealing for 1 min at 54°C. PCR products of the expected size were obtained and were successfully cloned into the TA cloning vector pCR2.1-TOPO (Invitrogen). Cloned amplicons were confirmed as correct by automated DNA sequencing, and a plasmid harboring the correct insert was designated pDJM385. The spectinomycin resistance marker from pSPECR [69] was excised with *Cla *I and cloned into the unique *Cla *I site (beginning at nucleotide 615 of the cloned 848-bp) of pDJM385 to yield pDJM386. Competent *H. influenzae *were transformed to spectinomycin resistance with pDJM386, using the static aerobic method as previously described [70], and selected on sBHI agar containing spectinomycin. Correct chromosomal recombinations were confirmed by the molecular size of a PCR product resolved on an agarose gel (data not shown).

### Growth Studies with *H. influenzae*

Growth studies were performed using the Bioscreen C Microbiology Reader (Oy Growth Curves AB Ltd., Helsinki, Finland) as previously described [19,71]. Briefly *H. influenzae *strains were inoculated from 12-14 hour cultures on chocolate agar with bacitracin into 10 ml of hdBHI and incubated for 4 h with shaking at 37°C. The 4 h cultures were pelleted by centrifugation, washed once in phosphate buffered saline (PBS) containing 0.1% w/v gelatin, and resuspended to an optical density at 605 nm of 0.5 in the same buffer. One ml of the bacterial suspension was diluted in 5 ml of the same buffer and this final bacterial suspension was used to inoculate media for growth curves (0.1% v/v inoculum to give an approximate initial concentration of 200,000 c.f.u. per ml).

### Growth conditions for iron/heme (FeHm) regulated gene expression

Growth conditions pertaining to the FeHm-regulation window of *H. influenzae *strains Rd KW20, 10810 and R2866 have been previously defined [49,50], and were used as the basis for growth of strain R2846. The primary inoculum of strain R2846 was prepared as previously [49,50] so as to yield a final concentration of ~2 × 10^7 ^cfu/ml when 5 ml of inoculum was added to 120 ml of growth medium. The kinetics of repression of genes of interest by FeHm were determined as follows. Two flasks were prepared and inoculated with the primary inoculum as described above. Both flasks contained FeHm-restricted media (i.e. hdBHI additionally supplemented with 150 μM deferroxamine to chelate iron). Samples were taken from both flasks at 30 minute intervals for RNA isolation and Q-PCR analysis. After 90 minutes of incubation, FeHm (0.5 mM FeCl_3_, 10 μg/ml heme) was added to one of the two flasks and samples were removed at 5 minute intervals from both flasks for RNA isolation. Broth cultures for iron and heme (FeHm) mediated regulation of gene expression were incubated in a rotary shaker at 175 rpm at 37°C. The samples removed for Q-PCR analysis were immediately mixed with RNAProtect (Qiagen, Valencia, CA) (500 μl samples mixed with 1 ml RNAProtect) and frozen at -70°C for later RNA preparation.

### RNA purification

Samples for Q-PCR obtained as described above were thawed, remixed by brief vortexing and incubated at room temperature for 5 minutes prior to purification using the RNeasy mini kit (Qiagen, Valencia, CA). Following purification, the sample was eluted with 40 μl of sterile RNase free water. Residual chromosomal DNA was removed by digestion with amplification grade DNase I (Invitrogen, Carlsbad, CA). The RNA samples were used to prepare cDNA as previously described [72]. Each 20 μl reaction contained 7 μl template RNA, 5.5 mM MgCl_2_, 500 μM each dNTP (dATP, dCTP, dGTP, dTTP), 1 × RT buffer, 80 mU RNase Inhibitor and 25 U MultiScribe Reverse Transcriptase (Applied Biosystems, Foster City, Ca.). The synthesis reaction was incubated at 25°C for 10 minutes followed by a further 30 minutes at 48°C. The reaction was terminated by heating at 95°C for 5 minutes. Prior to analysis, the cDNA was diluted by addition of 180 μl RNase-free water.

### Quantitative real-time PCR

Q-PCR was performed as previously described [72]. Gene-specific oligonucleotide primers (Table 4) were designed using Primer Express 2.0 (Applied Biosystems) and were tested to determine amplification specificity, efficiency and for linearity of the amplification with RNA concentration. A typical 25 μl reaction contained 12.5 μl of SYBR Green Master Mix, 250 nM of each primer, and 5 μl of cDNA sample. Quantification reactions for the target transcripts at each timepoint were performed in triplicate and normalized to concurrently run 16 s rRNA levels from the same sample. Relative quantification of gene expression was determined using the 2^-ΔΔCt ^method of Livak and Schmittgen where ΔΔC_t _= (C_t,Target _- C_t,16 s_)_Timex _- (C_t,Target _- C_t,16 s_)_Control _[73].

**Table 4:** Primers used for quantitative-PCR

**Primer**^**a**^	Sequence 5' to 3'
QPCR-16s-F	TCGTCAGCAAGAAAGCAAGCT

QPCR-16s-R	GCTGGCGGCAGGCTTAA

QPCR-adhC-F	CTGCTGAATGTGGCGAATGT

QPCR-adhC-R	CTGACCATCTGGCATTAAGC

QPCR-hxuC-F	CGAGGGTTAAGTGATAATCGTGTT

QPCR-hxuC-R	AGCTACTTGGTCCTTTGATTACTTCAATT

QPCR-fhuA-F	CCGTCGTTTCGGTGATAACAA

QPCR-fhuA-R	TCGTGATCAATTTCGCTTTCG

QPCR-fhuC-F	AATTAATCGGCATGGGACGTT

QPCR-fhuC-R	TTTATCCGCCGCCGTTT

^a ^Primer pairs used to assay for each gene. Primer pair QPCR-fhuA-F and QPCR-fhuA-R are used to assay transcriptional status of r2846.1777.

## Authors' contributions

All authors contributed to the design and execution of the experiments detailed. DJM constructed mutants and performed growth studies. EJT and PDH performed PCR analyses. TMV performed expression analyses. DJM drafted the manuscript. PWW, TWS and TLS revised the manuscript. All authors read and approved the final manuscript.

## References

[B1] Turk DC The pathogenicity of *Haemophilus influenzae* J Med Microbiol 1984 18 1 16 10.1099/00222615-18-1-1 6146721

[B2] Panek H O'Brian MR A whole genome view of prokaryotic haem biosynthesis Microbiology 2002 148 2273 2282 12177321 10.1099/00221287-148-8-2273

[B3] White DC Granick S Hemin biosynthesis in *Haemophilus* J Bacteriol 1963 85 842 850 14044953 10.1128/jb.85.4.842-850.1963 PMC278235

[B4] Schlor S Herbert M Rodenburg M Blass J Reidl J Characterization of ferrochelatase (*hemH*) mutations in *Haemophilus influenzae* Infect Immun 2000 68 3007 3009 10.1128/IAI.68.5.3007-3009.2000 10769004 PMC97519

[B5] Loeb MR Ferrochelatase activity and protoporphyrin IX utilization in *Haemophilus influenzae* J Bacteriol 1995 177 3613 3615 7768877 10.1128/jb.177.12.3613-3615.1995 PMC177073

[B6] Morton DJ Stull TL Crosa JH, Mey AR, Payne SM Haemophilus Iron Transport in Bacteria 2004 Washington, DC: American Society for Microbiology 273 292

[B7] Genco CA Dixon DW Emerging strategies in microbial haem capture Mol Microbiol 2001 39 1 11 10.1046/j.1365-2958.2001.02231.x 11123683

[B8] Griffiths E Bullen JJ, Griffiths E Iron in biological systems Iron and Infection: Molecular, Physiological and Clinical Aspects 1999 New York, NY: John Wiley & Sons, Inc 1 26

[B9] Ward CG Bullen JJ Bullen JJ, Griffiths E Clinical and Physiological Aspects Iron and Infection: Molecular, Physiological and Clinical Aspects 1999 New York, NY: John Wiley & Sons, Inc 369 450

[B10] Evans RW Crawley JB Joannou CL Sharma ND Bullen JJ, Griffiths E Iron proteins Iron and Infection: Molecular, Physiological and Clinical Aspects 1999 New York, NY: John Wiley & Sons, Inc 27 86

[B11] Peters T All About Albumin: Biochemistry, Genetics, and Medical Applications 1996 New York, NY: Academic Press

[B12] Stull TL Protein sources of heme for *Haemophilus influenzae* Infect Immun 1987 55 148 153 3025098 10.1128/iai.55.1.148-153.1987 PMC260293

[B13] Morton DJ VanWagoner TM Seale TW Whitby PW Stull TL Catalase as a source of both X- and V-factor for *Haemophilus influenzae* FEMS Microbiol Lett 2008 279 157 161 10.1111/j.1574-6968.2007.01020.x 18093136

[B14] Morton DJ VanWagoner TM Seale TW Whitby PW Stull TL Utilization of myoglobin as a heme source by *Haemophilus influenzae *requires binding of myoglobin to haptoglobin FEMS Microbiol Lett 2006 258 235 240 10.1111/j.1574-6968.2006.00230.x 16640579

[B15] Morton DJ Whitby PW Jin H Ren Z Stull TL Effect of multiple mutations in the hemoglobin- and hemoglobin-haptoglobin-binding proteins, HgpA, HgpB, and HgpC of *Haemophilus influenzae *type b Infect Immun 1999 67 2729 2739 10338475 10.1128/iai.67.6.2729-2739.1999 PMC96576

[B16] Seale TW Morton DJ Whitby PW Wolf R Kosanke SD VanWagoner TM Stull TL Complex role of hemoglobin and hemoglobin-haptoglobin binding proteins in *Haemophilus influenzae *virulence in the infant rat model of invasive infection Infect Immun 2006 74 6213 6225 10.1128/IAI.00744-06 16966415 PMC1695506

[B17] Morton DJ Seale TW Madore LL VanWagoner TM Whitby PW Stull TL The haem-haemopexin utilization gene cluster (*hxuCBA*) as a virulence factor of *Haemophilus influenzae* Microbiology 2007 153 215 224 10.1099/mic.0.2006/000190-0 17185550

[B18] Morton DJ Smith A VanWagoner TM Seale TW Whitby PW Stull TL Lipoprotein *e *(P4) of *Haemophilus influenzae *: Role in heme utilization and pathogenesis Microbes Infect 2007 9 932 939 10.1016/j.micinf.2007.03.013 17548224 PMC1975679

[B19] Morton DJ Madore LL Smith A VanWagoner TM Seale TW Whitby PW Stull TL The heme-binding lipoprotein (HbpA) of *Haemophilus influenzae *: role in heme utilization FEMS Microbiol Lett 2005 253 193 199 10.1016/j.femsle.2005.09.016 16289530

[B20] Herrington DA Sparling PF *Haemophilus influenzae *can use human transferrin as a sole source for required iron Infect Immun 1985 48 248 251 3872264 10.1128/iai.48.1.248-251.1985 PMC261943

[B21] Morton DJ Williams P Utilization of transferrin-bound iron by *Haemophilus *species of human and porcine origins FEMS Microbiol Lett 1989 53 123 127 10.1111/j.1574-6968.1989.tb03609.x 2533128

[B22] Pidcock KA Wooten JA Daley BA Stull TL Iron acquisition by *Haemophilus influenzae* Infect Immun 1988 56 721 725 2964410 10.1128/iai.56.4.721-725.1988 PMC259360

[B23] Holland J Towner KJ Williams P Isolation and characterisation of *Haemophilus influenzae *type b mutants defective in transferrin-binding and iron assimilation FEMS Microbiol Lett 1991 61 283 287 10.1111/j.1574-6968.1991.tb04362.x 2037233

[B24] Williams P Morton DJ Towner KJ Stevenson P Griffiths E Utilization of enterobactin and other exogenous iron sources by *Haemophilus influenzae*, *H. parainfluenzae *and *H. paraphrophilus *J Gen Microbiol 1990 136 2343 2350 2150414 10.1099/00221287-136-12-2343

[B25] Morton DJ Williams P Siderophore-independent acquisition of transferrin-bound iron by *Haemophilus influenzae *type b J Gen Microbiol 1990 136 927 933 2143216 10.1099/00221287-136-5-927

[B26] Schryvers AB Identification of the transferrin- and lactoferrin-binding proteins in *Haemophilus influenzae* J Med Microbiol 1989 29 121 130 10.1099/00222615-29-2-121 2543820

[B27] Krewulak KD Vogel HJ Structural biology of bacterial iron uptake Biochim Biophys Acta 2008 1778 1781 1804 10.1016/j.bbamem.2007.07.026 17916327

[B28] Andrews SC Robinson AK Rodríguez-Quiñones F Bacterial iron homeostasis FEMS Microbiol Rev 2003 27 215 237 10.1016/S0168-6445(03)00055-X 12829269

[B29] Schwyn B Neilands JB Universal chemical assay for the detection and determination of siderophores Anal Biochem 1987 160 47 56 10.1016/0003-2697(87)90612-9 2952030

[B30] Morton DJ Characterization of iron uptake mechanisms in *Haemophilus *species Ph.D. Thesis 1989 University of Nottingham, Department of Pharmaceutical Sciences

[B31] University of Washington Genome Center http://genome.wustl.edu/

[B32] Mikael LG Pawelek PD Labrie J Sirois M Coulton JW Jacques M Molecular cloning and characterization of the ferric hydroxamate uptake (*fhu*) operon in *Actinobacillus pleuropneumoniae *Microbiology 2002 148 2869 2882 12213932 10.1099/00221287-148-9-2869

[B33] Braun V Braun M Killmann H Crosa JH, Mey AR, Payne SM Ferrichrome- and citrate- mediated iron transport Iron Transport in Bacteria 2004 Washington, DC: American Society for Microbiology 158 177

[B34] Wiener MC TonB-dependent outer membrane transport: going for Baroque? Curr Opin Struct Biol 2005 15 394 400 10.1016/j.sbi.2005.07.001 16039843

[B35] Jung JJ Vu DM Clark B Keller FG Spearman P *Neisseria sicca*/*subflava *bacteremia presenting as cutaneous nodules in an immunocompromised host Pediatr Infect Dis J 2009 28 661 663 10.1097/INF.0b013e318196bd48 19483662

[B36] del Rio ML Navas J Martin AJ Gutierrez CB Rodriguez-Barbosa JI Rodriguez Ferri EF Molecular characterization of *Haemophilus parasuis *ferric hydroxamate uptake (*fhu*) genes and constitutive expression of the FhuA receptor Vet Res 2006 37 49 59 10.1051/vetres:2005039 16336924

[B37] Matzanke BF Bohnke R Mollmann U Reissbrodt R Schunemann V Trautwein AX Iron uptake and intracellular metal transfer in mycobacteria mediated by xenosiderophores Biometals 1997 10 193 203 10.1023/A:1018351728081 9243798

[B38] Cuiv PO Clarke P O'Connell M Identification and characterization of an iron-regulated gene, *chtA*, required for the utilization of the xenosiderophores aerobactin, rhizobactin 1021 and schizokinen by *Pseudomonas aeruginosa *Microbiology 2006 152 945 954 10.1099/mic.0.28552-0 16549659

[B39] Anderson MT Armstrong SK The *Bordetella *Bfe system: growth and transcriptional response to siderophores, catechols, and neuroendocrine catecholamines J Bacteriol 2006 188 5731 5740 10.1128/JB.00495-06 16885441 PMC1540089

[B40] Payne SM Mey AR Crosa JH, Mey AR, Payne SM Pathogenic *Escherichia coli*, *Shigella*, and *Salmonella* Iron Transport in Bacteria 2004 Washington, DC: American Society for Microbiology 199 218

[B41] Diarra MS Dolence JA Dolence EK Darwish I Miller MJ Malouin F Jacques M Growth of *Actinobacillus pleuropneumoniae *is promoted by exogenous hydroxamate and catechol siderophores Appl Environ Microbiol 1996 62 853 859 8975614 10.1128/aem.62.3.853-859.1996 PMC167851

[B42] National Center for Biotechnology Information http://www.ncbi.nlm.nih.gov/Genomes/

[B43] Wellcome Trust Sanger Institute http://www.sanger.ac.uk

[B44] Harrison LH Simonsen V Waldman EA Emergence and disappearance of a virulent clone of *Haemophilus influenzae *biogroup aegyptius, cause of Brazilian purpuric fever Clin Microbiol Rev 2008 21 594 605 10.1128/CMR.00020-08 18854482 PMC2570154

[B45] Musser JM Barenkamp SJ Granoff DM Selander RK Genetic relationships of serologically nontypable and serotype b strains of *Haemophilus influenzae* Infect Immun 1986 52 183 191 3485574 10.1128/iai.52.1.183-191.1986 PMC262217

[B46] Mikael LG Srikumar R Coulton JW Jacques M *fhuA *of *Actinobacillus pleuropneumoniae *encodes a ferrichrome receptor but is not regulated by iron Infect Immun 2003 71 2911 2915 10.1128/IAI.71.5.2911-2915.2003 12704168 PMC153255

[B47] Cope LD Yogev R Muller-Eberhard U Hansen EJ A gene cluster involved in the utilization of both free heme and heme:hemopexin by *Haemophilus influenzae *type b J Bacteriol 1995 177 2644 2653 7751272 10.1128/jb.177.10.2644-2653.1995 PMC176933

[B48] Kidd SP Jiang D Jennings MP McEwan AG Glutathione-dependent alcohol dehydrogenase AdhC is required for defense against nitrosative stress in *Haemophilus influenzae* Infect Immun 2007 75 4506 4513 10.1128/IAI.00487-07 17591795 PMC1951181

[B49] Whitby PW VanWagoner TM Seale TW Morton DJ Stull TL Transcriptional profile of *Haemophilus influenzae *: Effects of iron and heme J Bacteriol 2006 188 5640 5645 10.1128/JB.00417-06 16855256 PMC1540045

[B50] Whitby PW Seale TW VanWagoner TM Morton DJ Stull TL The iron/heme regulated genes of *Haemophilus influenzae *: Comparative transcriptional profiling as a tool to define the species core modulon BMC Genomics 2009 10 6 10.1186/1471-2164-10-6 19128474 PMC2627913

[B51] Whitby PW Sim KE Morton DJ Patel JA Stull TL Transcription of genes encoding iron and heme acquisition proteins of *Haemophilus influenzae *during acute otitis media Infect Immun 1997 65 4696 4700 9353052 10.1128/iai.65.11.4696-4700.1997 PMC175673

[B52] Speziali CD Dale SE Henderson JA Vines ED Heinrichs DE Requirement of *Staphylococcus aureus *ATP-binding cassette-ATPase FhuC for iron-restricted growth and evidence that it functions with more than one iron transporter J Bacteriol 2006 188 2048 2055 10.1128/JB.188.6.2048-2055.2006 16513734 PMC1428144

[B53] Pramanik A Braun V Albomycin uptake via a ferric hydroxamate transport system of *Streptococcus pneumoniae *R6 J Bacteriol 2006 188 3878 3886 10.1128/JB.00205-06 16707680 PMC1482914

[B54] Turner PC Thomas CE Stojiljkovic I Elkins C Kizel G Ala'Aldeen DA Sparling PF Neisserial TonB-dependent outer-membrane proteins: detection, regulation and distribution of three putative candidates identified from the genome sequences Microbiology 2001 147 1277 1290 11320131 10.1099/00221287-147-5-1277

[B55] Bernier G Girijavallabhan V Murray A Niyaz N Ding P Miller MJ Malouin F Desketoneoenactin-siderophore conjugates for Candida: evidence of iron transport-dependent species selectivity Antimicrob Agents Chemother 2005 49 241 248 10.1128/AAC.49.1.241-248.2005 15616301 PMC538862

[B56] Heymann P Gerads M Schaller M Dromer F Winkelmann G Ernst JF The siderophore iron transporter of Candida albicans (Sit1p/Arn1p) mediates uptake of ferrichrome-type siderophores and is required for epithelial invasion Infect Immun 2002 70 5246 5255 10.1128/IAI.70.9.5246-5255.2002 12183576 PMC128288

[B57] Schalk IJ Metal trafficking via siderophores in Gram-negative bacteria: specificities and characteristics of the pyoverdine pathway J Inorg Biochem 2008 102 1159 1169 10.1016/j.jinorgbio.2007.11.017 18221784

[B58] Caballero-Mellado J Onofre-Lemus J Estrada-de Los SP Martinez-Aguilar L The tomato rhizosphere, an environment rich in nitrogen-fixing *Burkholderia *species with capabilities of interest for agriculture and bioremediation Appl Environ Microbiol 2007 73 5308 5319 10.1128/AEM.00324-07 17601817 PMC1950987

[B59] Kang HY Brickman TJ Beaumont FC Armstrong SK Identification and characterization of iron-regulated *Bordetella pertussis *alcaligin siderophore biosynthesis genes J Bacteriol 1996 178 4877 4884 8759851 10.1128/jb.178.16.4877-4884.1996 PMC178270

[B60] Harris JK De Groote MA Sagel SD Zemanick ET Kapsner R Penvari C Kaess H Deterding RR Accurso FJ Pace NR Molecular identification of bacteria in bronchoalveolar lavage fluid from children with cystic fibrosis Proc Natl Acad Sci USA 2007 104 20529 20533 10.1073/pnas.0709804104 18077362 PMC2154465

[B61] Bittar F Richet H Dubus JC Reynaud-Gaubert M Stremler N Sarles J Raoult D Rolain JM Molecular detection of multiple emerging pathogens in sputa from cystic fibrosis patients PLoS One 2008 3 e2908 10.1371/journal.pone.0002908 18682840 PMC2483419

[B62] Barenkamp SJ Leininger E Cloning, expression, and DNA sequence analysis of genes encoding nontypeable *Haemophilus influenzae *high-molecular-weight surface-exposed proteins related to filamentous hemagglutinin of *Bordetella pertussis* Infect Immun 1992 60 1302 1313 1548058 10.1128/iai.60.4.1302-1313.1992 PMC256997

[B63] Fleischmann RD Adams MD White O Clayton RA Kirkness EF Kerlavage AR Bult CJ Tomb J Dougherty BA Merrick JM McKenney K Sutton G FitzHugh W Fields C Gocayne JD Scott J Shirley R Liu L Glodek A Kelley JM Weidman JF Phillips CA Spriggs T Hedblom E Cotton MD Utterback RC Hanna MC Nguyen DT Saudek DM Brandon RC Fine LD Fritchman JL Fuhrmann JL Geoghagen NSM Gnehm CL McDonald LA Small KV Fraser CM Smith HO Venter JC Whole-genome random sequencing and assembly of *Haemophilus influenzae *Rd Science 1995 269 496 512 10.1126/science.7542800 7542800

[B64] Nizet V Colina KF Almquist JR Rubens CE Smith AL A virulent nonencapsulated *Haemophilus influenzae *J Infect Dis 1996 173 180 186 8537657 10.1093/infdis/173.1.180

[B65] Bakaletz LO Kennedy B-J Novotnoy LA Duquesne G Cohen J Lobet Y Protection against development of otitis media induced by nontypeable *Haemophilus influenzae *by both active and passive immunization in a chinchilla model of virus-bacterium superinfection Infect Immun 1999 67 2746 2762 10338477 10.1128/iai.67.6.2746-2762.1999 PMC96578

[B66] Morton DJ Seale TW Bakaletz LO Jurcisek JA Smith A VanWagoner TM Whitby PW Stull TL The heme-binding protein (HbpA) of *Haemophilus influenzae *as a virulence determinant Int J Med Microbiol 2009 299 479 488 10.1016/j.ijmm.2009.03.004 19451029 PMC2749905

[B67] Rogers HJ Iron-binding catechols and virulence in *Escherichia coli* Infect Immun 1973 7 445 456 16558077 10.1128/iai.7.3.445-456.1973 PMC422698

[B68] Poje G Redfield RJ General methods for culturing *Haemophilus influenzae* Methods Mol Med 2003 71 51 56 12374030 10.1385/1-59259-321-6:51

[B69] Whitby PW Morton DJ Stull TL Construction of antibiotic resistance cassettes with multiple paired restriction sites for insertional mutagenesis of *Haemophilus influenzae *FEMS Microbiol Lett 1998 158 57 60 10.1111/j.1574-6968.1998.tb12800.x 9453156

[B70] Morton DJ Bakaletz LO Jurcisek JA VanWagoner TM Seale TW Whitby PW Stull TL Reduced severity of middle ear infection caused by nontypeable *Haemophilus influenzae *lacking the hemoglobin/hemoglobin-haptoglobin binding proteins (Hgp) in a chinchilla model of otitis media Microb Pathog 2004 36 25 33 10.1016/j.micpath.2003.08.007 14643637

[B71] Morton DJ VanWagoner TM Seale TW Whitby PW Stull TL Differential utilization by *Haemophilus influenzae *of hemoglobin complexed to the three human haptoglobin phenotypes FEMS Immunol Med Microbiol 2006 46 426 432 10.1111/j.1574-695X.2006.00052.x 16553817

[B72] VanWagoner TM Whitby PW Morton DJ Seale TW Stull TL Characterization of three new competence-regulated operons in *Haemophilus influenzae* J Bacteriol 2004 186 6409 6421 10.1128/JB.186.19.6409-6421.2004 15375121 PMC516621

[B73] Livak KJ Schmittgen TD Analysis of relative gene expression data using real-time quantitative PCR and the 2^-ΔΔC_T _^method Methods 2001 25 402 408 10.1006/meth.2001.1262 11846609

[B74] Alexander HE Leidy G Determination of inherited traits of *H. influenzae *by desoxyribonucleic acid fractions isolated from type-specific cells J Exp Med 1951 93 345 359 10.1084/jem.93.4.345 14824407 PMC2136084

[B75] Wilcox KW Smith HO Isolation and characterization of mutants of *Haemophilus influenzae *deficient in an adenosine 5'-triphosphate-dependent deoxyribonuclease activity J Bacteriol 1975 122 443 453 165169 10.1128/jb.122.2.443-453.1975 PMC246077

